# The profile of patients presenting with postpartum depression seen in the Department of Obstetrics and Gynaecology at Dr George Mukhari Academic Hospital

**DOI:** 10.4102/sajpsychiatry.v24i0.1310

**Published:** 2018-09-26

**Authors:** Bernadette M. Muluvhu, Solomon Rataemane

**Affiliations:** 1 Department of Psychiatry, Sefako Makgatho Health Science University, South Africa

## Abstract

**Introduction:**

Postpartum depression is one of the most common psychiatric complications of childbearing women. Most studies report that it has a high prevalence, but women at risk are rarely recognised during pregnancy and post-delivery. It is therefore often missed and, as a result, it has negative effects not only on the mother but also on the newborn infant.

The aim of this study was to determine the profile of patients presenting with postpartum depression seen in the wards and outpatients section of the Department of Obstetrics and Gynaecology at Dr George Mukhari Academic Hospital (DGMAH).

**Methods:**

A cross-sectional descriptive study was conducted where 150 consenting mothers at 6 weeks postpartum at the postnatal clinic were recruited from DGMAH using set inclusion and exclusion criteria. The participants were individually interviewed for socio-demographic characteristics. The Edinburgh Postnatal Depression scale, a self-screening scale for depression, was used to evaluate for the prevalence of depression.

**Results:**

A total of 150 postnatal mothers were interviewed. The prevalence of postpartum depression at 6 weeks postpartum at DGMAH was found to be 8.9%. The age range of participants was between 18 and 44 years, with a mean age of 28.70 years. The majority of participants (149, 99.3%) were black people, 112 (74.7%) were single and 119 (79.3%) were unemployed. The most common mode of delivery was caesarean section (92.0%, 138), 74 (49.3%) had unplanned pregnancies and 76 (50.7%) were unsatisfied with the infant gender. Psychosocial stressors (*p* = 0.003) and infant’s birth weight of 2.5 kg (*p* = 0.005) were statistically associated with postpartum depression.

**Conclusion:**

There is a high prevalence of postpartum depression in South Africa. A routine screening of mothers at the peri-partum period and early intervention is recommend to avoid its consequences on the mother, family and developing children.

